# Meeting of the General Nursing Council for England and Wales

**Published:** 1921-06-11

**Authors:** 


### REGISTRATION RULES IN PRINT SHORTLY.

### Meeting of the General Nursing Council for England and Wales.

The fifteenth meeting of the General Nursing Council for England and Wales was held at the Ministry of Health, Whitehall, on Friday, June 3. There were present: Mr. J. C. Priestley, K.C., Chairman of the Council, in the chair; Lady Hobhouse, Dr. E. W. Goodall, Dr. Bedford Pierce, Sir T. Jenner Verrall, Mr. T. Christian, Miss A. Coulton, Miss R. Cox Davies, Miss A. Dowbiggin, Mrs. Bedford Fenwick, Miss A. Lloyd Still, Miss M. MacCallum, Miss I. Macdonald, Miss M. E. Sparshott, Miss E. C. Suiss, and Miss Villiers. Apologies for non-attendance were reported from the Hon. Mrs. Eustace Hills, Miss M. Tuke, the Rev. G. B. Cronshaw, and Miss Seymour Yapp, the latter being prevented by illness. Miss Tuke regretted that owing to her inability to attend the Council meetings with regularity on account of the pressure of work she felt bound to resign her seat. Her resignation was accepted with regret, and thanks were voted for the services she has rendered to the Council. Miss Tuke is a nominee of the Board of Education, who will be asked to appoint another person.

The following letters were reported: (1) From the National Union of Trained Nurses, sending a resolution passed at the last annual meeting supporting the General Nursing Council in maintaining the standard of training and in insisting on a term for general training for all nurses to be admitted to the General Register: (2) from the Central Council of Infant and Child Welfare, reporting the appointment by the Central Committee for the Care of Cripples of a sub-committee to bring before the General Nursing Council the matter of the training and registration of nurses trained in an orthopædic hospital and passing a qualifying examination held under the auspices of the General Nursing Council. Should such be granted registration as orthopædic nurses? Further, should orthopædic nurses desiring general training in addition be given shortened training in general hospitals covering those branches of nursing which orthopædic hospitals are unable to provide? The Sub-Committee desired to meet representatives of the General Nursing Council on these points. The CHAIRMAN asked the meeting for their views, and pointed out that acceptance of the proposal would mean another register. Mrs. BEDFORD FENWICK strongly deprecated the establishment of further supplementary registers. The acceptance of such a suggestion would tend to chip away the value of the general training of nurses. No other profession is sub-divided in such a manner as proposed. She pointed out that the Council has under consideration the system of affiliated training and the grouping of hospitals, and orthopædic hospitals will come under that head. Miss SPARSHOTT agreed. She urged orthopædic nurses should be encouraged to take general training. Dr. GOODALL and Miss MACCALLUM both expressed their support of the previous speakers, and the CHAIRMAN undertook to convey to the Central Council for Infant and Child Welfare the Council's reply in this sense.

The opening ceremony of the Council's new premises at 12 York Gate, Regent's Park, has been graciously undertaken by H.R.H. Princess Christian, and will take place on Friday, June 10. The house will accommodate about 120 guests, and the Chairman submitted the list of those to be invited, which the Council discussed, amended, and passed.

### WHY THE RULES HAVE BEEN DELAYED.

Certain alterations in the rules already passed by the Council were brought forward by the Chairman of the Registration Committee for consideration and adoption. In presenting them, Mrs. BEDFORD FENWICK said that, to the Council's disappointment, the Ministry of Health has come to a determined conclusion that there must be reciprocity between the three Councils without an equivalent standard. The Chairman was in sympathy with the view that, following the example of Registration Councils throughout the world (of which there are fifty-seven), there should be inserted in the Rules an equivalent standard for registration. Scotland and Ireland objected to this, and the legal adviser of the English Ministry advised that the Council has no jurisdiction in the matter. Upon the Council contesting this, it was referred to the Law Officers of the Crown, who supported the Ministry. The Registration Committee remains unconvinced, but in the circumstances it has been necessary to redraft the rule dealing with reciprocal registration of nurses in England and Wales, Scotland and Ireland. It was sent to the Nursing Councils of the two latter countries and their opinion asked for. The Council's chief point is to ensure uniformity of qualification and uniformity of standard between the three countries before reciprocity can take place. No agreement has yet been come to with Scotland, but agreement with Ireland has been reached so far that the following recommendation was carried at the meeting on June 3: "That the Minister of Health be asked to sanction the opening of the Register in conjunction with that of Ireland." It was also resolved that the Rules as drafted shall now be printed, Sir JENNER VERRALL urging that there should be no further delay.

Certain rules dealing with fees were also passed. The fee for existing nurses reciprocally registered is to be £1 1s. for admission to one part of the Register, and 10s. 6d. for admission to any second, third, or subsequent part of the Register, and for intermediate nurses after the term of grace two guineas and one guinea.

The Scottish Council has raised the question of the definition of the term "Registered Nurse," to describe a nurse registered in the General part of the Register, desiring instead the term "Registered General Nurse" or any similar phrase. Mrs. Bedford Fenwick protested against this, and as Chairman of the Registration Committee stated that the term "Registered Nurse" would be adhered to.

### A CENTRAL PRELIMINARY TRAINING-SCHOOL.

The report of the Education and Examination Committee was presented by its Chairman, Miss Lloyd Still. It records that over 300 matrons, superintendent nurses, and tutor-sisters attended the informal Conference on April 28, and recommends two alterations in the draft Syllabus, which were agreed to. [These alterations were published in THE HOSPITAL of May 14, p. 122.] An important recommendation of the Education and Examination Committee is that it shall be empowered to construct a scheme of central preliminary training-schools for nurses as part of the prescribed training for admission to the Register for future nurses. It recommends that a list of approved hospitals to be recognised by the Council be compiled, and reports the circulation to the Chairman of general hospitals and the Clerk to the Guardians of Poor-law infirmaries of the statement that as a condition of the admission of any person to the Register the Council will require that that person shall have undergone the training prescribed in the Syllabus and the production of certified statements that she has been trained in the subjects laid down in the Syllabus and passed the examinations of the Council. The condition of inclusion in the Council's list of approved institutions is the adoption of the curriculum of training and direction that the evidence required shall be given. The Education and Examination Committee further recommends that, in order to show that the prescribed training has been given, a system should be adopted similar to that of medical schools, and the information conveyed to the Council by means of certificates certifying that the candidate has received training in the subjects laid down by the Council in the Syllabus. The report of the Education and Examination Committee was accepted.

Sir T. JENNER VERRALL presented the report of the Finance Committee. The accounts for payment were passed, including several in connection with furnishing the new headquarters. The grateful thanks of the Council were expressed to the three ladies, acting as a sub-committee, responsible for the furnishing—viz., Mrs. Bedford Fenwick, Miss Cox Davies, and Miss Villiers, the two latter of whom declared that to Mrs. Fenwick belonged the chief credit. The clerical staff at York Place will consist of a senior shorthand typist, a second shorthand typist, and a typist for stencilling and copying. The appointments to these posts were confirmed, as were also those on the domestic staff—viz., Miss Cameron, housekeeper; an assistant housekeeper, and two scrubbers. The Council gave authority that an accountant is to be selected and appointed temporarily.

